# Comparison of orthodontic treatment need and malocclusion prevalence according to KIG, ICON, and mIOTN in German 8- to 9-year-old children of the Sixth German Oral Health Study (DMS 6)

**DOI:** 10.1007/s00056-023-00446-6

**Published:** 2023-02-01

**Authors:** Christian Kirschneck, Kathrin Kuhr, Cristiana Ohm, Nicolas Frenzel Baudisch, Andreas Rainer Jordan

**Affiliations:** 1 Department of Orthodontics, University Hospital Regensburg, Franz-Josef-Strauß-Allee 11, 93053 Regensburg, Germany; 2 Institute of German Dentists, Universitätsstr. 73, 50931 Cologne, Germany

**Keywords:** Orthodontic treatment need, Malocclusion prevalence, Orthodontic Indication Groups, Index of Complexity, Outcome and Need, Modified Index of Orthodontic Treatment Need, Kieferorthopädischer Behandlungsbedarf, Prävalenz von Malokklusion, Kieferorthopädische Indikationsgruppen, Index of Complexity, Outcome and Need, Modifizierter Index of Orthodontic Treatment Need

## Abstract

**Purpose:**

The aim of the present study was to compare the malocclusion indices KIG (Kieferorthopädische Indikationsgruppen, Orthodontic Indication Groups), ICON (Index of Complexity, Outcome and Need), and mIOTN (modified Index of Orthodontic Treatment Need) regarding differences in malocclusion prevalence and their assessment of orthodontic treatment need in German 8‑ to 9‑year-old children of the Sixth German Oral Health Study (Deutsche Mundgesundheitsstudie, DMS 6).

**Methods:**

The necessary data for the calculation of the KIG, mIOTN, and ICON were collected by a dentist as part of a clinical orthodontic examination during the field phase of the DMS 6 and by a subsequent digital orthodontic model–analytical evaluation of intraoral scans of the dental arches and the occlusal situation in habitual occlusion.

**Results:**

Prevalence, severity, and treatment need of tooth and jaw misalignments differed in part considerably depending on the index used for assessment. On the other hand, there were several outcomes which yielded quite similar results for the different indices used, such as orthodontic treatment need, which ranged from 40.4% (KIG) over 41.6% (ICON) to 44.2% (mIOTN). Interestingly, orthodontic treatment need for the individual subject could differ considerably, when assessed using different indices.

**Conclusions:**

In general, the results show that the mIOTN is much more conservative in assessing malocclusion prevalences often being smaller than those derived by KIG or ICON. In contrast, KIG and ICON often yield similar prevalences with certain distinct differences due to discrepancies in the respective definitions and also clearly differentiate between treatment possibility and arbitrarily determined treatment need.

## Abstract (de)

**Ziel:**

Das Ziel der vorliegenden Studie war es, die Indizes KIG (Kieferorthopädische Indikationsgruppen), ICON (Index of Complexity, Outcome and Need) und mIOTN (modifizierter Index of Orthodontic Treatment Need) hinsichtlich Unterschieden in der Malokklusionsprävalenz und deren Einschätzung des kieferorthopädischen Behandlungsbedarfs bei deutschen 8‑ bis 9‑jährigen Kindern der Sechsten Deutschen Mundgesundheitsstudie (DMS 6) zu vergleichen.

**Methode:**

Die notwendigen Daten zur Berechnung von KIG, mIOTN und ICON wurden von einem Zahnarzt im Rahmen einer klinisch-kieferorthopädischen Untersuchung während der Feldphase der DMS 6 und durch eine anschließende digitale kieferorthopädische modellanalytische Auswertung von Intraoralscans der Zahnbögen und der okklusalen Situation in habitueller Okklusion erhoben.

**Ergebnisse:**

Prävalenz, Schweregrad und Behandlungsbedarf von Zahn- und Kieferfehlstellungen unterschieden sich je nach dem zur Bewertung herangezogenen Index zum Teil erheblich. Andererseits gab es mehrere Endpunkte, die für die verschiedenen verwendeten Indizes recht ähnliche Ergebnisse lieferten, wie etwa der kieferorthopädische Behandlungsbedarf, der von 40,4 % (KIG) über 41,6 % (ICON) bis 44,2 % (mIOTN) reichte. Interessanterweise konnte der kieferorthopädische Behandlungsbedarf des einzelnen Probanden erheblich variieren, wenn er anhand verschiedener Indizes bewertet wurde.

**Schlussfolgerungen:**

Im Allgemeinen zeigen die Ergebnisse, dass der mIOTN beim Assessment der Malokklusionsprävalenzen deutlich konservativer ist, sie waren oft geringer als beim Assessment mit KIG bzw. ICON. Dagegen ergeben sich nach KIG und ICON oft ähnliche Prävalenzen mit gewissen deutlichen Unterschieden aufgrund von Diskrepanzen in den jeweiligen Definitionen. KIG und ICON differenzieren auch klar zwischen Behandlungsmöglichkeit und willkürlich festgestelltem Behandlungsbedarf.

## Introduction

Misaligned teeth and jaws are among the most common health problems affecting the oral cavity, along with caries and periodontal diseases [2]. The primary task of orthodontics is the preventive and corrective treatment and elimination of malfunctions as well as tooth and jaw misalignments with pathological value [15]. This includes the detection, prevention, diagnostics and therapy of malformations of the masticatory system, as well as tooth position and bite anomalies, jaw malformations and deformations of the jaw and the facial skull [12]. Orthodontic abnormalities are also associated with limitations in chewing, breathing, phonetics, and swallowing [12]. In this sense, orthodontics is a preventive discipline if treatment can prevent secondary diseases [15]. The causes of orthodontic anomalies are multifactorial and range from genetic, epigenetic, and functional to environmental factors. The degree of severity of the individual diseases is also extremely variable. The treatment options are correspondingly extensive. Genetic and epigenetic factors are difficult to influence through orthodontic therapy; treatment is primarily directed against the consequences or the phenotypic manifestation. In the case of functional and environmental factors, on the other hand, there are fundamentally preventive options and often a causal therapy option [12].

Current, population-wide data on the prevalence of tooth and jaw misalignments and corresponding orthodontic treatment need in Germany are not available. The last nationwide recording dates from 1989 being the First German Oral Health Study (DMS1) [13]. In particular, there are no systematic epidemiological data on tooth and jaw misalignments from the new federal states. This means that the overall orthodontic and epidemiological picture in Germany is not complete—with corresponding uncertainties for the planning of dental health care, a gap that has now been closed with the current Sixth German Oral Health Study (Deutsche Mundgesundheitsstudie, DMS 6), which for the first time in over 30 years aimed to quantify prevalence, severity and treatment need of tooth and jaw misalignments in the general German population of 8‑ to 9‑year-old children.

Prevalence, severity, and treatment need of tooth and jaw misalignments can be quantified by means of various epidemiological indices, which have been specifically developed for this purpose over the years. Since the Orthodontic Indication Groups (Kieferorthopädische Indikationsgruppen, KIG) represent and reflect the orthodontic care provided by dentists in Germany in statutory health insurance, they were included in the DMS 6 as a leading index for sociopolitical reasons. In Germany, the Orthodontic Indication Groups are a diagnosis-related classification scheme for assessing reimbursement of orthodontic treatment services within the framework of contractual dental care provided by statutory health insurance [1, 17]. On January 1, 2002, KIG replaced the therapy-oriented indication system that had been in use until then. Malocclusions of the patient are categorized into eleven etiological groups and assigned to one of five degrees of severity. Statutory health insurance in Germany covers payment for orthodontic treatment, if the degree of severity reaches grade 3 in at least one etiological group [1, 17].

The Orthodontic Indication Groups are based on the Index of Orthodontic Treatment Need (IOTN) index, which is used in an analogous manner to assess orthodontic treatment need in Great Britain by the National Health Service (NHS) [2]. IOTN is an internationally well-established index and has been used as epidemiological tool in various studies before [3, 5]. To allow international comparability of results, we also aimed to assess the IOTN as part of the DMS 6—as concerns regarding its complexity, the need for a longer training period, and its reliability in epidemiological studies have been raised; however, we decided to calculate the modified version of the IOTN (mIOTN), which was specifically developed for oral health surveys [6].

ICON (Index of Complexity, Outcome and Need) is probably the most suitable index for epidemiological investigations [11] and was therefore also assessed as part of the DMS 6. The ICON index was developed by Daniels and Richmond (the developers of the Peer Assessment Rating Index, PAR) in 2000 [7] and is based on a consensus process of 97 orthodontists from eight European countries and the USA, which represents a significant advantage over other indices, as ICON is validated across Europe and the USA. The validity of the index has been shown in several studies [8, 16]. It represents an improvement of the PAR index, as it reassesses the individual occlusal parameters in terms of their importance, takes aesthetic aspects into account and, in addition to assessing the treatment result, also enables an assessment of the need for treatment, similar to the Index of Orthodontic Treatment Need (IOTN) [4]. Studies have shown that the ICON can replace the PAR, the Dental Aesthetic Index (DAI) and the IOTN [9], as it takes into account not only the treatment outcome, but also the severity of the anomaly initially present. It can also be used efficiently clinically, since it can be derived in a short time per case using both jaw models and clinical assessments [11].

As prevalence, severity, and treatment need of tooth and jaw misalignments were assessed by three different epidemiological indices in the context of the DMS 6 (KIG, mIOTN, and ICON), it is reasonable to surmise that outcomes will differ, as different criteria for classifying malocclusion prevalence, severity, and treatment need exist for KIG, mIOTN, and ICON. The aim of the present study in the framework of the Sixth German Oral Health Study (DMS 6) was therefore to compare the malocclusion indices Orthodontic Indication Groups (Kieferorthopädische Indikationsgruppen, KIG), ICON, and mIOTN regarding differences in malocclusion prevalence and their assessment of orthodontic treatment need in German 8‑ to 9‑year-old children of the Sixth German Oral Health Study (DMS 6).

## Materials and methods

The DMS 6 is an oral epidemiological examination and social science survey on a nationally representative level with a focus on tooth and jaw misalignments. The investigations took place from January–March 2021 in 16 study centers in Germany. After an address drawing in the municipal administrations of the study centers, 1892 people from the birth cohorts of 2011 and 2012 were invited to take part in the study. A total of 714 study participants were dentally examined and socially questioned. All relevant data were available for 705 study participants and included in the statistical analysis. The response rate was 40.6%, and 51.4% of the study participants were male (female: 48.6%), the proportion of 8‑year-old children was 49.4% (9-year-olds: 50.6%). A survey of nonrespondents was then conducted to gain insight into any systematic differences between study participants and nonstudy participants. Since the analysis did not show any systematic differences between the study participants and the nonstudy participants surveyed, no distortion of the study results can be assumed due to the proportion of nonrespondents and the study results can be regarded as representative.

The necessary data for the calculation of the KIG, mIOTN, and ICON were collected, on the one hand, by a dentist as part of a clinical orthodontic examination during the field phase of the DMS 6 and, on the other hand, by the subsequent digital orthodontic model–analytical evaluation of intraoral scans of the dental arches and the occlusal situation in habitual occlusion. Habits, dyskinesias, and dysfunctions were recorded, on the one hand, by questioning the study participants and, on the other hand, by a dental diagnosis. Craniofacial anomalies, such as cleft lip and palate, were also recorded as part of the dental diagnosis.

For reasons of research ethics, a comprehensive X‑ray examination as part of the DMS 6 was not possible. Tooth retention, tooth displacement, hyper- and hypodontia, as they are recorded according to KIG, can only be reliably detected with the help of radiation-invasive methods. In a purely clinical study, the prevalences would probably be underestimated. For this reason, the above findings were not collected. For further details regarding the methodology of the DMS 6, please refer to the methods paper of the DMS 6 [10].

### KIG

The assessment of the Orthodontic Indication Groups (Kieferorthopädische Indikationsgruppen, KIG) was carried out as described in the guidelines of the Federal Committee of Dentists and Health Insurance Companies for orthodontic treatment in the version dated June 4, 2003 and published on September 24, 2003 in the Federal Gazette No. 226 (p. 24966) dated December 3, 2003 [1], supplemented by the content presented in the monograph “Orthodontic Accounting” [17]. In contrast to clinical practice, not only the highest degree of severity was recorded, i.e., not only the category with the highest score was documented, but the degree of severity was determined and recorded separately for each of the etiological groups, since a study participant could also have several different types of malocclusions of different degrees of severity. Orthodontic treatment need is present in cases of severity degrees 3, 4, and 5 according to the regulations of the statutory health insurance in Germany [1].

### mIOTN

The modified Index of Treatment Need (mIOTN) was calculated as described in the literature [6, 14]. The mIOTN consists of two components. The aesthetic component IOTN-AC was determined as part of the clinical orthodontic examination using a standardized series of images; it is identical to the aesthetic component in the Index of Complexity, Outcome and Need (ICON). The dental component includes 5 malocclusions: missing teeth, overjet, crossbites, displacement of contact points (crowding), and overbite. The aim of mIOTN is to determine a definite need for orthodontic treatment. There are no further classifications according to severity/complexity. In a first step, it is determined for each component or each malocclusion whether there is a definite need for treatment. Only if no need is determined for any of the components, a subject is assigned the category “No need for treatment”.

### ICON

The Index of Complexity, Outcome and Need (ICON) was evaluated as described in the literature [7, 9]. As with the orthodontic indication groups (KIG), not only the highest degree of severity was recorded, i.e., not only the category with the highest score value, but the degree of severity was determined and recorded separately for each of the 7 groups, since a study participant could also have several different types of malocclusions and degrees of severity. The aesthetic component ICON-AC, which is identical to the assessment of the aesthetic component of the IOTN-AC, was determined using a standardized questionnaire. In order to determine the total score, the severity of the 7 malocclusion groups is multiplied by a respective weighting factor and the values obtained are added up to the actual ICON index value (weighted total score, range 1–122). If the total score is greater than 43, treatment according to ICON is mandatory. In addition, the ICON index was used to assess the complexity of the treatment.

## Results

### Orthodontic treatment need

According to KIG, orthodontic treatment need corresponding to KIG degrees 3, 4, and 5, was found in 40.4% (*N* = 285) of surveyed German children 8–9 years old. According to ICON, treatment need in the same population corresponding to a total ICON score greater than 43 was determined to be 41.6% (*N* = 278) and according to mIOTN 43.3% (*N* = 305) only considering the dental component and 44.2% (*N* = 312) also considering the aesthetic component of mIOTN. The mean value of the aesthetic component was 3.2 points. Since treatment according to ICON is indicated from a total score of 44 points, it is possible due to the weighting factor that an indication for treatment is triggered solely by the aesthetic assessment of the teeth, without further clinical findings having to be available. This is the case from an aesthetic rating of “7” (out of 10). This affected 2.5% of the study participants. For KIG aesthetic evaluations are irrelevant and not considered.

**Fig. 1:**
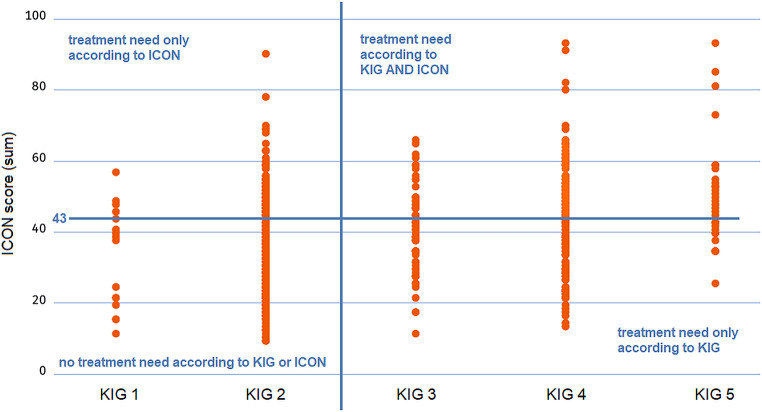
Scatterplot depicting subjects categorized into different KIG grades and corresponding ICON scores. Orthodontic treatment need (and severity of malocclusion) is assessed differently by both epidemiological indices for the individual subject, although the orthodontic treatment need determined for the total population is quite similar (KIG 40.4%, ICON 41.6%). *KIG* orthodontic treatment need (Kieferorthopädische Indikationsgruppen), *ICON* Index of Complexity, Outcome and Need Scatterplot zur Einteilung der Probanden in verschiedene KIG-Grade und entsprechende ICON-Scores. Der kieferorthopädische Behandlungsbedarf (und Schweregrad der Malokklusion) wird von den beiden epidemiologischen Indizes für den einzelnen Probanden unterschiedlich bewertet, obwohl der für die Gesamtpopulation ermittelte kieferorthopädische Behandlungsbedarf relativ ähnlich ist (KIG 40,4%, ICON 41,6%). *KIG* Kieferorthopädische Indikationsgruppen, *ICON *Index of Complexity, Outcome and Need

A scatter plot shows that subjects, who have been identified to be in need of orthodontic treatment by one index, are not necessarily assessed the same way by another index, as seen in a comparison of KIG and ICON (Fig. 1). Ideally, one would expect a linear dependency in which, for example, all ICON scores in the grade 1 KIG group are also in the lower range, at least not exceeding the limit score of 43/44 points. However, this is not the case. The same applies to KIG grade 5: If the indices would yield congruent results, it would be expected in this case that the ICON scores would all be beyond the absolute treatment indication of 44 points. This is not the case either. The evaluation shows that the intersection, in which both indices indicate a treatment indication (upper right quadrant) is only 46.6%.

### Severity/complexity of malocclusion

Severity of malocclusion is expressed in KIG by different KIG degrees from 1–5. This is somewhat mirrored in ICON by the complexity of malocclusion treatment, which is also expressed in five degrees from “easy”, “mild”, “moderate” over “difficult” to “very difficult”. Severity or complexity of malocclusion is not considered in mIOTN. KIG degrees 1, 2, 3, 4, and 5 were found in 2.5% (*N* = 18), 57.0% (*N* = 402), 10.0% (*N* = 70), 25.5% (*N* = 180), and 5.0% (*N* = 35) of the study population, respectively, and ICON complexities “easy”, “mild”, “moderate”, “difficult” and “very difficult” in 22.1% (*N* = 148), 57.8% (*N* = 386), 16.6% (*N* = 111), 1.8% (*N* = 12), and 1.7% (*N* = 11).

### Craniofacial anomalies

Craniofacial anomalies, which is predominantly the presence of oral clefting, were found in 0.4% (*N* = 3) of the study population according to KIG, whereas ICON and mIOTN do not include this assessment.

### Buccal and lingual nonocclusion

Buccal and lingual nonocclusion was found in 0.3% (*N* = 2) of the study population according to KIG, whereas ICON and mIOTN do not include this assessment.

### Distal and mesial malocclusion

Distal malocclusion is assessed in KIG according to the degree of sagittal dental overjet of incisors and categorized in three degrees of severity with degrees 2, 4 and 5 corresponding to an increased overjet of 3–6 mm, 6–9 mm and greater than 9 mm, respectively. Mesial malocclusion is determined by the degree of reverse overjet with degree 4 corresponding to a reverse overjet up to 3 mm and degree 5 over 3 mm. mIOTN on the other hand considers only an overjet of greater than 6 mm as distal malocclusion and a reverse overjet of greater than 3.5 mm as mesial malocclusion (if masticatory or speech anomalies are present, mesial malocclusion is already considered from a reverse overjet of 1 mm onwards). By contrast, ICON does not rely on sagittal dental overjet in this assessment, but rather considers occlusion in the buccal segment with any cusp relation deviating from cusp to embrasure as malocclusion not differentiating between mesial and distal malocclusion. Distal malocclusion according to KIG (degrees 2, 4, and 5) was found in 88.9% (*N* = 621) of surveyed German children 8–9 years old and mesial malocclusion (degrees 4 and 5) in 4.0% (*N* = 28). According to mIOTN, prevalence of distal malocclusion was 19.7% (*N* = 137), which corresponds to KIG degrees 4 and 5 combined, and of mesial malocclusion 0.6%. According to ICON mesial and distal malocclusion combined amounted to 76.5% (*N* = 512) at the left and 77.7% (*N* = 519) at the right jaw side.

### Dental crowding

Dental crowding according to KIG is assessed separately for the anterior (category E) and posterior (category P) segments of the dental arch with three degrees of severity 2, 3, and 4, respectively (degree 2 corresponding to mild crowding of > 1 mm [anterior segment] up to 3 mm, degree 3 moderate crowding up to 4 mm in the posterior segments and 5 mm in the anterior segment and degree 4 corresponding to severe crowding exceeding 4 mm or 5 mm in the posterior or anterior segments). mIOTN does not differentiate between anterior and posterior segments and considers any proximal contact point deviation of 4 mm or above between neighboring teeth as dental crowding. ICON also does not differentiate between anterior and posterior segments defining 5 degrees of crowding with degrees 1, 2, 3, 4, and 5 corresponding to 2.1–5 mm, 5.1–9 mm, 9.1–13 mm, 13.1–17 mm, and > 17 mm (or impacted teeth, which could not be assessed in this study) of crowding, respectively, but only considering crowding in the upper dental arch. Crowding according to ICON and in the posterior segments according to KIG is determined by comparing the sum of the mesiodistal crown diameters to the respectively available arch length, whereas crowding according to mIOTN and in the anterior segment according to KIG is defined via proximal contact point deviations. Anterior dental crowding according to KIG was found in 60.8% (*N* = 428) of German children 8–9 years old (mild/moderate/severe in 51.6%, 8.4%, and 0.7%, respectively) and posterior dental crowding in 29.2% (*N* = 206) of children (mild/moderate/severe in 23.5%, 3.1%, and 3.6%, respectively). According to mIOTN prevalence of dental crowding was 4.0% (*N* = 28). According to ICON dental crowding was present in 6.9% (*N* = 46) of the study population with degrees 1, 2, and 3 found in 5.7%, 1.0%, and 0.2%, respectively, and degrees 4 and 5 not found at all.

### Dental spacing

Dental spacing is not assessed by KIG and mIOTN indices, but only by ICON and only in the upper dental arch differentiating three degrees of spacing with degrees 1, 2, and 3 corresponding to 2–5 mm, 5–9 mm, and > 9 mm of spacing. Prevalence in the study population according to ICON was 68.7% (*N* = 459) with degrees 1, 2, and 3 contributing 32.2%, 29.1%, and 7.4%, respectively.

### Crossbite

KIG differentiates three types of posterior crossbite, namely cusp-to-cusp bite (degree 2), bilateral (degree 3), and unilateral crossbite (degree 4) with prevalences in the study population being 2.7% (*N* = 19), 0.4% (*N* = 3), and 5.3% (*N* = 37), respectively, amounting to a total prevalence of transversal malocclusions of 8.4% (*N* = 59). mIOTN on the other hand defines crossbite as a forced bite, i.e., a discrepancy between retruded contact position and intercuspal position of more than 2 mm, which was found in 23.0% of children (*N* = 162), also considering anterior crossbites, which indicate a mesial occlusion rather than a transversal problem. ICON follows the same principle as KIG defining any transverse relationship of cusp to cusp or worse as crossbite with the prevalence determined as 11.6% (*N* = 78), but also considers anterior crossbites. The definition of the crossbite according to mIOTN and ICON, which pool transversal and sagittal traits, does thus not correspond to the crossbite definition of KIG, which only considers the posterior crossbite.

### Open bite

Open bite according to KIG is defined as a vertical gap between incisal edges or cusps of upper and lower anterior or posterior teeth of up to 1 mm (degree 1), more than 1 mm (degree 2), 2 mm (degree 3), or 4 mm (habitual aetiology: degree 4, skeletal aetiology: degree 5). mIOTN only considers open bite from a vertical gap of 4 mm onward (corresponding to KIG degrees 4 and 5) as open bite, whereas ICON severity grading corresponds to the KIG system, except that no differentiation is made between habitual and skeletal aetiology (both classified as degree 4) and that only anterior open bite is considered by ICON. Open bite according to KIG was found in 7.1% (*N* = 50) of surveyed German children 8–9 years old (degrees 2, 3, and 4 in 4.6%, 1.6%, and 1.0%, respectively—degree 1 could not be assessed). According to mIOTN, prevalence of open bite was 1.0% (*N* = 7) and according to ICON open bite was present in 12.4% (*N* = 83) of the study population with degrees 1, 2, 3, and 4 found in 5.4%, 4.5%, 1.5%, and 1.0% of subjects, respectively.

### Deep bite

Deep bite according to KIG is defined as an increased vertical overlap between incisal edges of upper and lower anterior teeth of more than 3 mm (degree 2) or more than 3 mm with traumatic contact of incisal edges to the gingiva of the antagonist jaw (degree 3). mIOTN only considers KIG degree 3 as deep bite, whereas ICON defines deep bite as lower incisor coverage greater than one third (degree 1), two thirds (degree 2), or full coverage and beyond (degree 3). Deep bite according to KIG was found in 61.0% (*N* = 420) of German children 8–9 years old (degrees 2 and 3 in 51.2% and 9.8%, respectively). According to mIOTN prevalence deep bite was 9.8% (*N* = 67) and according to ICON 76.8% (*N* = 513) with degrees 1, 2, and 3 contributing 57.3%, 18.7%, and 0.8%.

## Discussion

We could confirm our hypothesis that prevalence, severity, and treatment need of tooth and jaw misalignments as assessed by the three epidemiological indices differed in part considerably depending on the index used for assessment. On the other hand, there were several outcomes which yielded quite similar results for the different indices used, such as orthodontic treatment need, which ranged from 40.4% (KIG) to 44.2% (mIOTN). This shows that despite the different composition of the international ICON and mIOTN indices with regard to malocclusions and components, but also weighting factors considered, an almost identical orthodontic treatment need was determined compared to the Orthodontic Indication Groups (KIG). In an international comparison, this finding confirms that the German KIG system can be regarded as a valid and interchangeably useable instrument for determining the need for orthodontic treatment. Furthermore, the KIG system and the orthodontic treatment need derived is in concordance with orthodontic treatment need as determined by other international indices, suggesting that the KIG system does not cause an over- or undersupply regarding orthodontic treatment delivered in the German population. This is supported by various previous studies on children from different European countries—a European international comparison with the available data shows that the orthodontic treatment need of 40.4% (KIG) to 44.2% (mIOTN), which was determined in the present study for the German population of 8–9 year olds, is European average. In an Estonian study [18], a significantly increased treatment need of 64.3% was indicated. On the other hand, a Croatian study reported a need for orthodontic treatment for 34% in children in the mixed dentition phase [20]. For the examined age group of 8- to 9‑year-old children, there are only a few studies available for a comparison, so that the study results can only be classified in the international context to a limited extent.

Interestingly, although orthodontic treatment need for the study collective in general was determined to be quite similar across the various indices, for the individual subject it was not. As Fig. 1 clearly shows, some subjects rated with no treatment need by one index had a treatment need when assessed by another index and vice versa. This is certainly due to the fact that different definitions, weightings, and demarcation points for the minimal severity of the respective type of malocclusion requiring treatment were rather arbitrarily determined for the individual indices and are not based on actual epidemiological data regarding the effects or functional–medical benefits of orthodontic treatment, when administered for different initial severities and types of malocclusion, which should be a focus of future research, although some findings in this regard are already available in the literature [12].

Although severity of malocclusion as determined by KIG and complexity of treatment as determined by ICON are not directly comparable, as they assess different entities, a certain comparison is possible, as a higher severity of malocclusion in consequence leads to a higher complexity of treatment, as suggested by direct comparison of mild malocclusion severity (KIG) and mild treatment complexity (ICON), which showed similar prevalences of about 57%. Interestingly, prevalence of treatment complexity assessed as “difficult” and “very difficult” by ICON was considerably less than prevalence of severe and very severe malocclusion according to KIG (degrees 4 and 5), indicating that also severe malocclusions can be effectively treated orthodontically without extreme difficulty.

Prevalence of craniofacial anomalies such as oral clefting and buccal and lingual nonocclusions was only assessed by KIG and can therefore not be compared between indices. Furthermore, due to the low prevalence found in the study population (0.3–0.4%), no valid epidemiological generalizations can be made.

Distal malocclusion, i.e., Angle class II, was found in 88.9% of subjects according to KIG and only 19.7% according to mIOTN, whereas ICON pools this assessment with mesial occlusion (with prevalence rates of about 4.0% according to KIG) [7], thus, yielding slightly biased prevalences of 76.5% and 77.7% for the left and right jaw sides. These distinctly differing results can be easily explained by the different demarcation point used for the extent of sagittal overjet supposed to require orthodontic treatment, which is set at > 3 mm by mIOTN [6] and > 6 mm by KIG [1], thus, yielding a lower prevalence for distal malocclusion for mIOTN. As ICON uses a completely different assessment of not optimal intercuspidation of antagonist teeth in the posterior dental arch, which includes any deviation from the ideal neutral occlusion [7], prevalence rates are quite high and similar to those derived by KIG, as the KIG system also defines distal occlusion starting from any deviation from normal overjet of 3 mm as degree 2 [17]. A quite similar situation is evident for mesial occlusion with prevalences determined as 4.0% by KIG and 0.6% by mIOTN with ICON not enabling this assessment. As in the KIG system any reverse overjet present is already classified as mesial occlusion [1], this is only the case for reverse overjets of > 3.5 mm (with exceptions if functional problems are present), thus, explaining the considerably lower prevalence of mesial occlusion according to mIOTN [6].

A similar situation is present for dental crowding with prevalence according to mIOTN (4.0%) and ICON (6.9%) being much smaller than according to KIG (60.8% anterior, 29.2% posterior crowding). As mIOTN only considers contact point deviations larger than 4 mm as crowding [6], which is quite extensive, prevalence is correspondingly low, whereas KIG considers all proximal contact point deviations larger than 1 mm in the anterior segment as dental crowding, thus, yielding the significantly higher prevalence rate [1]. As ICON assesses dental crowding via a comparison of mesiodistal crown widths and available arch length across the entire dental arch [9], differently than mIOTN or KIG, this might explain the significantly lower prevalence compared to the anterior segment according to KIG, but also the posterior segment, as KIG only considers mesiodistal crown widths and available arch length of the orthodontic support zone (canine and premolars) separately for each quadrant. Furthermore, dental crowding is mostly more pronounced in the lower dental arch due to tertiary crowding occurring at the lower incisors, which could also contribute to the lower prevalence found by ICON, which only assessed the upper dental arch [7]. As dental spacing is only assessed by ICON, a comparison to KIG and mIOTN cannot be made. Interestingly, prevalences of the different degrees of dental spacing were quite high reaching 68.7% in total—a fact that indicates that this malocclusion despite its high prevalence is not adequately reflected and considered by the KIG and ICON indices.

Prevalence of crossbite was found to be quite different for KIG (8.4%), mIOTN (23.0%), and ICON (11.6%). This is mainly due to completely different definitions of crossbite according to the different indices. Whereas KIG and ICON consider cusp-to-cusp bite, unilateral and bilateral crossbite morphologically, mIOTN follows a functional definition with crossbite defined as forced bite, i.e., a discrepancy between retruded contact position and intercuspal position of more than 2 mm [6], which does not necessarily correspond to the static bite situation in habitual occlusion. Furthermore, mIOTN and ICON both also consider a reverse overjet, which is a sagittal trait, whereas KIG defines crossbite as a transversal problem. Prevalence of crossbite according to ICON is thus approximately 4% (prevalence of reverse overjet according to KIG) higher than crossbite prevalence according to KIG. Furthermore, the definition of crossbite according to ICON also encompasses buccal and lingual nonocclusion, which are categorized separately in KIG.

Open bite prevalence according to KIG (7.1%) differed considerably from that assessed by mIOTN (1.0%) and ICON (12.4%). As discussed before, mIOTN is much stricter in the definition of open bite only considering vertical gaps of 4 mm and beyond as open bite [6], whereas KIG and ICON already consider open bites as any vertical gap present [1, 7], explaining the higher prevalence rates found. Prevalence according to ICON was still higher than that according to KIG most likely due to the fact that slight open bites of up to 1 mm (degree 1) could not be separately assessed by KIG and are thus missing in the KIG prevalence, which is thus slightly underestimated compared to ICON.

When considering deep bite prevalences, KIG (61.0%) and ICON (76.8%) yielded distinctly higher prevalences than mIOTN (9.8%). Again, mIOTN only designates quite extensive deep bites > 3 mm as such with contact of incisal edges to the gingiva of the antagonist jaw [6], whereas KIG considers any deep bite > 3 mm regardless of traumatic gingival contact present or not, thus, explaining the higher prevalence found according to KIG [1]. The highest deep bite prevalence was found for ICON, which is most likely due to the different definition of deep bite, as not an absolute value (such as 3 mm) for overbite is used in the assessment according to ICON, but rather the coverage of lower incisors by upper incisors being greater than one third of the labial surface [7], which may be less than the demarcation of 3 mm used by KIG and mIOTN depending on the relative height of the lower incisors.

A methodological limitation of the ICON and mIOTN indices is the fact that neither was developed for early mixed dentition, but they were rather developed for permanent (adult) dentition. In particular, when assessing the aesthetic component of the indices (AC) using a chart of ten orthodontic anomalies of increasing severity, there are problems in the transferability of the results, since the chart only shows the permanent dentition, which is not completely reliably transferrable to the 8‑ and 9‑year-old children. Since the aesthetic component is weighted by a factor of 7 in the ICON index, there is a certain potential for bias here. Another shortcoming is the fact that tooth retention, tooth displacement, hyper- and hypodontia could not be assessed within the scope of the DMS 6, because no X‑ray images were available for ethical reasons. However, it was clinically recorded whether a space maintainer or a replaced tooth (removable, e.g., children’s prosthesis) was present and whether a tooth was in semi-retention. Another limitation on the methodological side is the use of the Orthodontic Indication Groups (KIG) as an epidemiological index in a population of 8‑ to 9‑year-old children, while these are used to determine the reimbursement of orthodontic services within the framework of statutory health insurance for a population of > 10-year-olds. There is also a risk of underestimating the actual prevalence and orthodontic treatment need that arise 1–2 years later in the studied population aged > 10 years, since it is known that most orthodontic anomalies have a tendency to be aggravated during growth [19].

## Conclusions

In general, the results show that the mIOTN (modified Index of Orthodontic Treatment Need) is much more conservative in assessing malocclusions with prevalences often being smaller than those derived by KIG (Kieferorthopädische Indikationsgruppen, Orthodontic Indication Groups) or ICON (Index of Complexity, Outcome and Need). The reason for this is the fact that the mIOTN is a very simplified index that does not consider various severities of malocclusions, but was rather developed to only differentiate dichotomously between treatment need or no treatment need. In contrast, KIG and ICON often yield similar prevalences with certain distinct differences (e.g., for dental crowding) due to discrepancies in the respective definitions, how a certain type and severity of malocclusion is assessed and graded. Both KIG and ICON also clearly differentiate between treatment possibility and arbitrarily determined treatment need, as both indices define most malocclusions as any deviation from the norm (degree 2 or more in KIG, degree 1 or more in ICON, possibility for treatment) and only later apply demarcation points and recommendations, which severity actually requires treatment (treatment need), although these demarcation points were not derived based on epidemiological data, but rather clinical expertise and consensus. All patients not meeting requirements for treatment need, but having a malocclusion according to KIG/ICON degrees 2 and 1, thus, also presumably have the chance to profit from orthodontic treatment by correcting the malocclusion present, which is considered by KIG and ICON, but not by mIOTN.
